# Advances in the Application of Nanocomposite Hydrogels in Crops

**DOI:** 10.3390/gels11120957

**Published:** 2025-11-28

**Authors:** Diego Gael Hernández-Echave, Gonzalo Casillas-Moreno, Andrés Isaí Romo-Galindo, Tonantzin Anahí Gutiérrez-Gómez, Gilberto Velázquez-Juárez, Moyses Alejandro Rodríguez-Ortega, Rubén Octavio Muñoz-García, Diego Alberto Lomelí-Rosales

**Affiliations:** Departamento de Química, Universidad de Guadalajara, Blvd. Marcelino García Barragán 1421, Guadalajara 44430, Mexicogilberto.velazquez@academicos.udg.mx (G.V.-J.);

**Keywords:** nanocomposite hydrogels, water retention, biodegradable polymers, crops

## Abstract

Conventional agricultural practices, based on intensive irrigation and heavy fertilizer and pesticide inputs, are increasingly incompatible with climate change, soil degradation, and sustainability goals. Hydrogels have emerged as promising soil amendments to improve water and nutrient management, and fall broadly into two categories: synthetic polyacrylate/polyacrylamide-based systems and natural biobased hydrogels derived from polysaccharides such as alginate, cellulose, and chitosan. The latter, often obtained from agro-industrial residues, offer biodegradable and potentially lower-impact alternatives to persistent synthetic matrices. This review analyzes recent advances in the design and application of nanocomposite hydrogels in agricultural crops, with emphasis on high-value systems such as tomato, chili pepper and maize. Representative studies show that hydrogel–nanofertilizer formulations can increase soil water retention in tomato from ~55–56% to ~78–79%, nearly double swelling capacity in wheat, reduce irrigation requirements by around 15% in legumes, and improve plant biomass by ~30–40% under drought conditions. In parallel, nanocomposite hydrogels loaded with micronutrients, phytochemicals or biostimulants can enhance nutrient uptake, provide 36–80% protection against *Fusarium* wilt, and reduce postharvest pathogen growth by up to ~90%, while in some cases improving the nutraceutical quality of fruits. These outcomes illustrate a dual mechanism of action in which the hydrogel matrix acts as a micro-reservoir that buffers water and nutrients, whereas nano- and phytochemical components operate as physiological eustressors that modulate plant defense and metabolism. Finally, we discuss environmental and translational challenges, including hydrogel biodegradation pathways, the long-term fate and ecotoxicity of released nanoparticles, regulatory uncertainty, and market and field acceptance. Addressing these gaps through integrative agronomic, ecotoxicological, and regulatory studies is essential to ensure that nanocomposite hydrogels evolve into truly sustainable smart carriers for fertilizers, pesticides, and biostimulants in future cropping systems.

## 1. Introduction

Global challenges related to food security, water scarcity, and environmental degradation have driven the transition toward more sustainable and resilient agricultural systems. These issues have intensified the need to develop agricultural technologies capable of improving resource-use efficiency while minimizing ecological impact. Conventional agricultural practices, heavily dependent on excessive fertilizer application, pesticide use, and intensive irrigation, are increasingly unsustainable and incompatible with emerging climate constraints, soil degradation, and a rapidly growing global population [1].

In this context, the development of new functional materials capable of optimizing resource use and reducing environmental impact has become a scientific priority. Among the emerging strategies, polymer-based hydrogels have gained increasing attention due to their remarkable water-retention capacity, soil-conditioning properties, controlled release of agrochemicals, and improved nutrient-use efficiency [2]. Their biocompatibility, biodegradability, and ability to modify their chemical structures make them promising platforms for various agricultural applications, including moisture retention, sustained nutrient release, and reduced irrigation frequency [3].

Over the past decade, several review articles have synthesized advances in hydrogel science, addressing physicochemical characteristics, synthesis routes, and broad applications in agriculture. Notable contributions include comprehensive reviews on nanocomposite hydrogels [4], hydrogel-based controlled release systems [5], polymer–nanoparticle interactions, and smart materials designed for soil improvement. These studies have established solid conceptual foundation by describing general mechanisms of swelling behavior, nutrient release, and polymer–nanomaterial compatibility.

More recently, the incorporation of nanomaterials into the polymer matrix has led to the emergence of nanocomposite hydrogels, a new generation of hybrid materials with improved physicochemical and mechanical properties, with enhanced physicochemical, mechanical, and stimulus-responsive properties [6]. These materials synergistically combine the benefits of conventional hydrogels with the unique properties of nanomaterials, such as metal nanoparticles, nanocellulose, graphene, or nanoclays. Their incorporation enables improved environmental stability, antimicrobial activity, and finely tuned release profiles of agrochemicals, thereby expanding their potential in precision agriculture [7,8].

Despite these advances, significant gaps remain in the current literature. Many studies focus predominantly on material design and physicochemical characterization, offering limited insights into crop-specific responses, field-level performance, and the dual mechanisms through which these hybrid systems manage water while modulating physiological stress induced by nanostructured components [9]. Although substantial advances have been reported separately in hydrogel development and agricultural nanotechnology, few studies effectively integrate both perspectives to explain how these hybrid systems influence yield, nutraceutical quality [10], drought tolerance, or long-term soil health [11]. Moreover, the environmental implications of nanoparticle release and hydrogel biodegradation [12] while acknowledged—continue to raise unresolved questions related to ecotoxicity, regulatory frameworks [13,14], and alignment with sustainable agricultural transitions.

These advances have opened up new possibilities in the field of agricultural nanotechnology, where nanocomposite hydrogels can act as smart vectors for the efficient and environmentally safe delivery of fertilizers, pesticides, or biostimulants [15]. However, significant challenges remain, such as assessing their biodegradability, the potential toxicity of nanocomponents, and the economic viability for large-scale adoption, especially in low-resource agriculture [16,17].

Therefore, this review article aims to analyze recent advances in the design, synthesis, and application of nanocomposite hydrogels in agricultural crops, emphasizing their role in improving water and nutritional efficiency, increasing abiotic stress tolerance, and sustainable release of agrochemicals. Additionally, it addresses current limitations, environmental considerations, and future prospects in the development of smart hydrogel-based materials, geared towards truly sustainable agriculture.

## 2. Natural and Synthetic Hydrogels

Hydrogels, as innovative functional polymer materials, are viewed as promising agricultural tools due to their water-retaining capacity, swelling properties, controlled release, biocompatibility, and biodegradability [18,19,20,21]. These 3D polymer structures in agriculture are mainly applied for the controlled release of fertilizers, thereby improving nutrient use efficiency and reducing losses [22]. They also serve as soil conditioners with high water retention capacity, being used to prevent drying and cracking, particularly in arid soils [23]. Additionally, they are used for the sustained release of pesticides and other agrochemicals, contributing to reduced application frequency and environmental impact [24].

In agriculture, hydrogels are broadly classified into synthetic and natural polymers. Common synthetic types include polyacrylamide (PAM), crosslinked to synthesize superabsorbent polymers; polyacrylic acid (PAA) and sodium polyacrylate, valued for water retention and soil conditioning; and polyvinyl alcohol (PVA), noted for swelling capacity and biocompatibility. These characteristics are advantageous because it grants them long-term soil stability, allowing a single application to remain effective as a water reservoir for an extended period [23,25,26].

Conversely, natural polymer hydrogels comprise cellulose derivatives (e.g., carboxymethyl cellulose) for nutrient release, chitosan from crustacean shells for biocompatible blends, and alginate from seaweed for fertilizer and bioactive encapsulation. These are considered a more eco-friendly and sustainable solution because they break down over time into harmless, naturally occurring residues [24]. While their water-holding capacity might be slightly less than that of their synthetic counterparts, their ability to decompose ensures they do not accumulate in the soil, which aligns with goals for environmental preservation and soil health [27,28].

### 2.1. Synthetic Hydrogels in Crops

The application of synthetic hydrogels in modern agriculture is well-documented and extends beyond simple water retention to encompass resource management, soil enhancement, and stress mitigation [29]. Synthetic hydrogels, mainly based on polyacrylates (PAA) and polyacrylamides (PAM), maintain water in the soil for longer periods, thereby increasing plant growth and enhancing drought tolerance [3]. They can improve water infiltration, reduce surface runoff, and enhance soil physicochemical properties by improving soil structure and aggregate stability. In addition, they reduce evaporation losses by 20–30% by maintaining high soil moisture, which is crucial for healthy crop growth in water-limited areas, and help to minimize soil erosion, particularly in furrow and sprinkler irrigation systems [30].

However, these agronomic benefits come with important environmental concerns. The widespread use of synthetic polymers in various products has significantly contributed to global plastic pollution, and superabsorbent polymers (SAPs) such as PAA and PAM represent another class of man-made materials with uncertain long-term environmental consequences [31]. When these SAPs are introduced into the soil, they are susceptible to weathering and are shown to gradually lose their intended properties despite their designed durability. This prolonged exposure and decomposition in the environment can ultimately lead to the formation of persistent microplastic-like solid residues within the soil structure [32].

Because most synthetic hydrogels are non-biodegradable, their long-term persistence, potential toxicity of residual monomers (e.g., acrylamide), and limited cost-effectiveness for small-scale farmers have raised concerns and stimulated ongoing research into biodegradable alternatives [7,16,33]. These limitations have intensified the search for alternative water-retention and delivery systems that combine agronomic benefits with a lower environmental footprint.

### 2.2. Natural Biobased Hydrogels

In direct contrast to synthetic hydrogels, the pursuit of sustainable agricultural inputs has driven intensive research into natural biobased hydrogels. These materials, derived from renewable biopolymers such as starch, cellulose, alginate, and chitin/chitosan, are characterized by their biocompatibility and biodegradability, making them attractive candidates for environmentally responsible soil amendments. Their capacity to decompose into harmless residues reduces the risk of long-term accumulation in soils and better aligns with the goals of environmentally friendly and regenerative agriculture.

Cellulose-based gels, in particular, have found significant application in modern agriculture due to their versatile properties, which make them valuable components in the development of advanced and eco-friendly technologies [34]. Although plant-derived cellulose is not commonly used in hydrogel preparation in its native form because of its limited solubility in common solvents, it can be dissolved in specific solvent systems and transformed into hydrogels via physical or chemical cross-linking methods. These cellulose-derived networks, together with alginate- and chitosan-based hydrogels, offer promising routes to design materials that deliver water and nutrients while minimizing long-term accumulation in soils.

As summarized in Table 1, natural biobased hydrogels and synthetic hydrogels exhibit distinct differences in composition, performance, and environmental behavior. This comparison highlights the need for developing hybrid or nanocomposite hydrogel systems that integrate the advantages of both types, reducing environmental impacts while maintaining high agronomic efficiency.

**Table 1:** General comparison between natural biobased hydrogels and synthetic hydrogels used in agricultural applications [20,22,23,24,26,35].

Feature	Natural Biobased Hydrogels	Synthetic Hydrogels
Primary Composition	Natural Polymers (e.g., Starch, Cellulose, Chitosan, Alginate)	Petroleum-based Polymers (e.g., Polyacrylamide/PAM, Polyacrylates)
Key Advantage	Environmental Sustainability (Eco-Friendly)	Long-Term Performance & Durability
Biodegradability	Biodegradable (Decompose naturally into harmless residues)	Non-Biodegradable (Persist in the environment for many years)
Water Absorption Capacity	High, but often lower than synthetic (Typical range: 100–500 g/g).	Extremely High (Typical range: 400–1500 g/g).
Soil Stability/Longevity	Shorter duration; needs more frequent re-application as they decompose.	Excellent longevity; a single application can last for several seasons/years.
Environmental Impact	Very Low Impact. Their degradation can even release soil-enriching compounds and feed beneficial microbes.	Concerns over potential accumulation, especially if containing traces of monomers like acrylamide.
Reported Benefits to Crops	Excellent for enhancing seed germination and restoring degraded soils (as degradation products enrich the soil).	Highly effective for maximizing water retention and reducing irrigation frequency in arid conditions.

## 3. Functionalization of Hydrogels: Loading Systems

### 3.1. Metal Oxide Nanoparticles

From a mechanical standpoint, one of the most promising areas of innovation in hydrogel development is the incorporation of metal and metal oxide nanoparticles into the polymer network. Multiple studies have shown that these fillers act as additional physical cross-linking points, reinforcing the internal microstructure of the hydrogel and markedly increasing its strength, stability, and resistance to deformation [36]. For example, hydrogels containing TiO_2_ and CeO_2_ nanoparticles exhibited tunable textural properties, including more than a twofold increase in the spreadability index for TiO_2_-loaded systems and a 2.1-fold reduction in fresh matter loss in cultivated cabbage when CeO_2_-containing hydrogels were applied, with water retention extended by approximately two weeks compared with the control hydrogel [36,37]. Although metal nanoparticles can reduce the overall loading capacity of the matrix, they simultaneously enhance mechanical robustness and durability, improving the practical applicability of the material [38,39].

Beyond these mechanical improvements, metal-based nanoparticles also provide hydrogels with new functional properties. Depending on their composition and surface modification, they can introduce self-healing behavior, light-responsiveness, or antimicrobial activity into otherwise passive polymer networks. For instance, Au and Ag nanoparticles functionalized with azobenzene and azotriazole have been used to endow hydrogels with UV-triggered self-repair capability while maintaining the enhanced strength and stability conferred by nanoparticle reinforcement [38,39]. Such systems exemplify how nanoscale fillers can transform conventional hydrogels into dynamic, responsive materials.

These mechanical and functional enhancements support a range of specific applications. In the biomedical field, metal nanoparticle-containing hydrogels have been developed as antibacterial films and wound dressings that act as physical barriers, help prevent infection, and accelerate tissue recovery [40]. In agriculture, metal oxide nanoparticle–hydrogel composites have been explored as soil conditioners and moisture-management tools that reduce fresh matter loss and prolong favorable water availability in the rhizosphere, thereby improving crop performance under stress [36,37]. Overall, the integration of metal oxide nanoparticles into hydrogels illustrates how tailored nanofillers can simultaneously strengthen the polymer network, add functional responsiveness, and broaden the application space of these materials.

### 3.2. Phytochemicals

One way to functionalize hydrogels is by adding components to them. Due to their ability to store various compounds, hydrogels have been used in a variety of ways. One example is the introduction of phytochemicals into the structure of hydrogels. These compounds show great potential for application in different areas, such as medicine. For instance, *Artemisia dracunculus* essential oil, when integrated into the hydrogel matrix, conferred antimicrobial properties that may be useful in biological applications, proving effective against Gram-positive and negative bacteria [41]. Another example of the functionalization of hydrogels through the incorporation of phytochemical-rich extracts is the use of antioxidants and bioactive molecules present in *Tagetes erecta*, which, when incorporated into protective films on wounds, increased their ability to promote the healing of affected tissue and sustained antimicrobial activity for 7 days [42].

Another area where the use of hydrogels functionalized with bioactive compounds from plant tissues is rapidly gaining ground is the cosmetics industry, where it has been shown that the use of hydrogels loaded with plant extracts such as *Cannabis sativa* had positive effects on the inflammatory response of the skin, thus having a high value for application in various skin conditions thanks to their demonstrated ability to inhibit the activity of metalloproteinases in skin cells, as well as having a high presence of antioxidant compounds that can help protect against oxygen radical species [43].

In agriculture, microencapsulated hydrogels with phytohormones have also been used. Through the controlled release of these hormones, they have served as adaptation agents, triggering responses in plants that help them survive in difficult environments and extreme conditions, thus achieving a higher survival rate and ultimately higher production of the plants in question [44]. The use of phytochemicals in agriculture is not limited to increasing the adaptability of plants. Glycoalkaloids isolated from the Solanaceae family have also been used to load hydrogels for the disinfection of crop soils, proving effective in controlling fungal infections and thus helping to maintain optimal health in vegetables [45].

### 3.3. Nanoencapsulated Biostimulants

Hydrogels have shown immense potential for releasing compounds in different environments, one of these scenarios being the release of biostimulants to increase vegetable production. These biostimulants are classified as compounds that increase resistance to pathogens or adverse conditions and, as a result, stimulate plant growth and production [46]. One of the advances that has been achieved in hydrogels is the use of alginate for the microencapsulation of pyroglutamic acid. This organic acid has proven useful in increasing drought resistance in vegetables, although it has the disadvantage of being easily degraded in the soil. This is why microencapsulation in alginate hydrogels has increased its effectiveness up to tenfold, protecting the compound from direct contact with the harsh conditions, allowing it to be released more effectively and in the best conditions possible [47].

In this same vein, attempts have been made to nanoencapsulate other types of stimulants within hydrogels in order to apply them more efficiently. A successful case was the nanoencapsulation of sodium bisulfite medianone in chitosan to increase the drought resistance of *Solanum lycopersicon* L., significantly increasing the recovery rate of plants after severe water stress [48]. Chitosan has also been used as an insecticide by nanoencapsulating Beauvericin and applying it against *Spodoptera litura*, proving highly effective thanks to the stability acquired by the protein when nonencapsulated. Another advantage was the low environmental risk posed by the use of this type of nanoencapsulated insecticide due to its ability to be completely biodegradable, as well as its specificity [49]. Just as biopolymer hydrogels have been used for the nanoencapsulation of insecticidal agents used as biostimulants, porous silica gels have been used to microencapsulate orange oil, which has been surprisingly effective in controlling *Spodoptera littoralis*, considering the very low number of microspheres applied. This represented a great advantage because, as the orange oil is contained in the silica gel, greater physical resistance is achieved, it is very easy to apply, and it does not pose an environmental risk thanks to the inert nature of the silica microspheres [50].

### 3.4. Encapsulation and Controlled-Release Strategies

Encapsulation in hydrogels is a technique used in different areas of science and industry to protect and direct compounds that can be easily degraded or absorbed in the environment where they are found. The aim is to prevent them from being destroyed before performing the function for which they were designed. This encapsulation allows the compounds, extracts, or loads to be specifically directed to specific locations within an organism or environment so that they can be used optimally [51]. In the same way that hydrogel encapsulation protects sensitive loads, these encapsulations within the polymeric material allow for their controlled release by modifying the physical properties of the hydrogel that contains them. This is achieved through different mechanisms, one of which is the homogeneous distribution of the load within the polymer matrix, releasing the compounds as the gel loses its mechanical properties due to the action of temperature, pH, or external forces. Another release mechanism is the relaxation of the polymer networks, freeing the compounds to interact with the environment without compromising the polymer structure. In this mechanism, control is often achieved using physical barriers that prevent the pores in the hydrogel from opening completely, thus achieving partial or prolonged release of the load [36,52,53].

There are many examples of controlled-release hydrogels. In the food industry, the encapsulation of unstable phenolic and aromatic compounds protects them from premature degradation in films or gels so that they are released effectively once the food is consumed [21]. In the pharmaceutical industry, hydrogels have been used extensively for drug delivery thanks to their ability to be modified to respond to physiological stimuli. Dual hydrogels with polysaccharides have been developed that respond to changes in pH, releasing the antimicrobial drug baicalin at different rates depending on the pH to which they are exposed [52]. This release can be adjusted depending on the composition of the hydrogel, greatly increasing its applicability. In addition to drug delivery, hydrogels with the ability to release encapsulated loads are useful in the formulation of agrochemicals. Hydrogels composed of microalgae and polysaccharides have been developed that, by encapsulating urea in multilayer systems, have achieved prolonged release over several days thanks to weak intermolecular interactions that keep the urea adhered and encapsulated between the hydrogel caps, increasing the lifespan of fertilizers and their availability in the soil, making their use cheaper and reducing losses due to leaching to a minimum [54].

## 4. Dual Mechanism of Action in Crops

The effectiveness of nanocomposite hydrogels in agriculture is based on a dual mechanism of action, which combines the physical and chemical advantages of polymeric and nanoscale components. On the one hand, the hydrogel matrix functions as a micro-reservoir of water and nutrients, while the nanoparticles provide chemical functionality by enhancing micronutrient availability and introducing nanoscale reactivity. Together, this system prolongs root–soil interaction and increases the efficiency of water and nutrient uptake, thereby improving drought tolerance and productivity in degraded soils [3].

**Figure 1:**
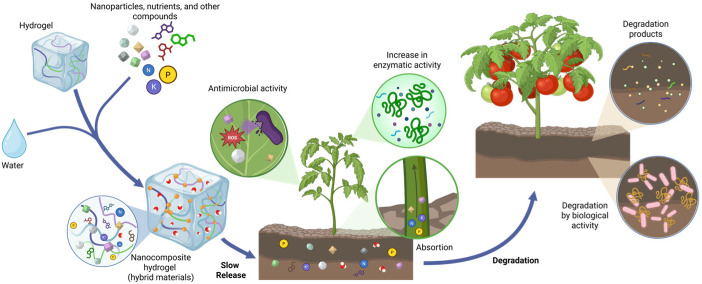
Schematic representation of the dual mechanism of action of nanocomposite hydrogels in crops. At the soil–root interface (left), the polymeric hydrogel matrix acts as a micro-reservoir that absorbs and stores water and dissolved nutrients, reduces percolation and evaporation losses, and releases agrochemicals in a controlled manner, thereby improving water-use and fertilizer-use efficiency. At the plant level (right), encapsulated nanoparticles, phytochemicals, and biostimulants function as physiological eustressors, triggering antioxidant defenses, hormonal rebalancing, and the accumulation of bioactive metabolites. The combined action of these physical and physiological processes leads to enhanced germination, growth, yield stability, and nutraceutical quality under abiotic and biotic stress conditions.

To better illustrate the dual mechanism proposed in this review, Figure 1 summarizes how nanocomposite hydrogels operate simultaneously at the soil and plant levels. This schematic integrates water and nutrient buffering in the hydrogel matrix with the physiological eustress elicited by nano- and phytochemical components, linking material properties to agronomic responses in crops.

As an example, the study by Marambio et al. highlights the potential of functional polymeric hydrogels as controlled-release matrices responsive to pH variations. Although the work focuses on herbicide release, the polymer architecture can be extrapolated to agro-biostimulant or nanoparticle-loaded systems, supporting the dual-action (i.e., controlled nutrient delivery and induced physiological eustress) [55].

Table 2 summarizes representative studies in which natural and synthetic hydrogels have been applied to major crops to improve water availability, nutrient-use efficiency, and overall growth performance. Across tomato, maize, and *Capsicum* spp., both biopolymer-based matrices (alginate, cellulose, pectin, starch) and synthetic systems (potassium polyacrylate, PVP/CMC) consistently mitigated abiotic stress by increasing soil water retention and enhancing plant performance indicators such as germination, plant height, leaf number, and chlorophyll content. Several formulations also acted as controlled-release matrices for urea or NPK fertilizers, prolonging nutrient availability while reducing leaching losses. Overall, these results indicate that both bio-based and synthetic hydrogels are effective platforms to improve water and fertilizer management in field-relevant crops, supporting their use as core components in sustainable soil–plant systems.

On the other hand, phytochemical-based nanoparticles or nanoconjugates act as physiological eustressors, capable of inducing mild and beneficial stress responses in plants. These nanoscale stimuli can activate antioxidant pathways, modulate hormonal balance, and stimulate the biosynthesis of secondary metabolites, resulting in increased stress tolerance, yield stability, and improved nutraceutical quality of crops [56,57]. This synergistic interaction between the hydrogel matrix and nanocomponents represents a breakthrough in the development of smart agricultural materials that optimize plant physiology through both passive and active mechanisms [9].

Recent applications in high-value crops—such as tomato (*Solanum lycopersicum*), chili pepper (*Capsicum* spp.), and corn (*Zea mays*)—have demonstrated significant improvements in plant growth, water use efficiency, fruit yield, and bioactive compound accumulation when using nanocomposite hydrogels [58,59]. Other reported cases cover horticultural and cereal systems, indicating broad applicability in various agricultural contexts. These findings highlight the potential of nanocomposite hydrogels as multifunctional agricultural technologies, capable of sustaining productivity under stress conditions while simultaneously improving the nutritional and functional quality of crops [19,60].

Table 3 compiles studies in which polymeric hydrogels are combined with nanoparticles, phytochemicals, or biostimulants to couple controlled release with physiological stimulation in crops. Most reports focus on tomato, where chitosan-, alginate-, starch-, and lignin-based hydrogels loaded with Cu, ZnO, or carbon nanostructures improve yield, fruit quality, nutrient uptake, and antioxidant status, while also conferring antifungal or nematicidal protection. In maize, gelatin-, lignin-, and CMC-based hydrogels containing nanofillers such as carbon dots or CaCO_3_ enhance germination, biomass accumulation, and drought tolerance, whereas in *Capsicum* systems, nanocomposite hydrogels and green-synthesized ZnO nanoparticles increase flowering, fruit yield, capsaicin content, and resistance to wilt diseases. Additional formulations tested in other crops demonstrate extended urea release, improved soil water retention, and early growth promotion. Taken together, these results suggest that nanocomposite and biostimulant-loaded hydrogels can simultaneously deliver agrochemicals and elicit beneficial stress responses, offering multifunctional tools to optimize crop productivity and resilience.

Taken together, the studies compiled for tomato, chili pepper, maize, wheat, rice, quinoa, soybean, lettuce, and other horticultural species reveal several recurring patterns across crops. First, nanocomposite hydrogels are most frequently applied to high-value horticultural crops (particularly Solanaceae and leafy vegetables), where they consistently improve water-use efficiency, biomass accumulation, and quality traits, often under irrigated or greenhouse conditions. Second, cereal crops exposed to drought (maize, wheat, rice) mainly benefit from enhanced soil water retention and macronutrient delivery, with yield and biomass gains closely linked to the swelling behavior and mechanical stability of the matrix. Third, legumes and pseudocereals (e.g., mung bean, quinoa) show pronounced responses at early developmental stages—germination, seedling establishment, and plant density—suggesting that nanocomposite hydrogels may be especially effective when targeted to critical windows of stand establishment. Across all crop groups, formulations based on chitosan, alginate, and acrylate-type polymers combined with ZnO-, Fe-, and Cu-based nanoparticles dominate the literature and consistently translate into improved stress tolerance and, in many cases, reduced water and agrochemical inputs.

**Table 2:** Representative studies using natural and synthetic hydrogels applied in crops such as maize, tomato and chili to improve water availability, fertilizer use efficiency, and basic plant growth performance.

Material	Species	Application	Findings	Ref.
Sodium alginate-based hydrogel.	Tomato (*Solanum Lycopersicon*)	Mitigation of water stress and prolonged release of Nitrogen (urea).	Yields increased by 19.58–43.18%. N release up to 15 days (86%). Improved germination rate, increased number of leaves, chlorophyll content, stem diameter, and plant height (better stress tolerance). No phytotoxicity was observed.	[61]
Biopolymer nanomaterials (cellulose, pectin, and starch).	Tomato (*Lycopersicon esculentum*)	Reduces the negative effects of salinity on tomato yield and quality.	Retention for both soluble and exchanged sodium ions. Increased tomato yield and mitigated salinity stress by enhancing antioxidant responses (phenols, flavonoids, and key antioxidant enzymes, including catalase and peroxidase).	[62]
Cross-linked potassium polyacrylate hydrogel.	Corn (*Zea mays*)	Mitigation of water stress.	Increase in available soil water by 49%, increase in water use efficiency from 13% to 41% for sandy soil and from 35% to 67% for loamy clay soil; increase in corn growth compared to control.	[3]
Hydrogels based on polyvinylpyrrolidone (PVP)/carboxymethyl cellulose (CMC) loaded with MPK ^1^ and NPK ^2^ fertilizers.	Corn (*Zea mays*)	Mitigation of water stress and prolonged release of NPK and MPK fertilizers.	Retention and prolonged release of fertilizers for up to 9 days. Reduction in nutrient leaching.	[63]
Cellulose microfiber hydrogel (CMH) from sugarcane bagasse.	Chilli (*Capsicum annuum*)	Mitigation of water stress and improvement in plant growth.	Greater average plant height was observed at concentrations of 0.5 and 2% (56.74 ± 2.51% and 58.06 ± 3.02%, respectively), as well as a greater number of leaves (18.67–11.33%).	[64]

^1^ Monopotassium-phosphate, ^2^ Nitrogen-phosphate-potassium.

**Table 3:** Studies involving polymeric hydrogels embedded with nanoparticles, phytochemicals or biostimulatns that provide systems that combine controlled release with physiological stimulation (eustress) to improve crop yield, antioxidant responses and fruit nutraceutical quality.

**Tomato**				
**Material**	**Species**	**Application**	**Findings**	**Ref.**
Hydrogel + Carbon Nanoparticles	Tomato (*Solanum lycopersicum*)	Improvement of soil biological properties and mitigation of water stress	Better growth of tomato plants (in terms of plant morphological parameters), 22–45% increase in growth, 16–29% increase in nutritional indices (P, Fe, and Zn), and 89% increase in microbial population.	[9]
Hydrogel + NC-MMt ^1^ (calcium montmorillonite)	Tomato, cv. ‘BRS Nagai’ *(Solanum lycopersicum)*	Improved seedling growth	Application at 1.5% improved the surface area and volume of tomato seedling roots with no toxic effects observed.	[65]
PVA-chitosan-CuNPs (Copper nanoparticles) complex	Tomato (*Solanum lycopersicum*)	Eustress treatment, fruit improvement	Increased yield by 60.68%, fruit number by 35.99%, fruit weight by 18.2%, and root dry/fresh weight by 80.87%. It also enhanced defense responses by raising PAL ^2^ activity (369.23%) and overexpressing PR1 ^3^ gen.	[66]
Chitosan-based hydrogel + Copper nanoparticles (CuNPs)	Tomato (*Solanum lycopersicum)*	Evaluation of plant growth and antioxidant content	Improved tomato growth, yield, and nutritional traits, increasing clusters by 11%, fruits by 29%, fruit weight by 25%, fresh root weight by 20%, and dry weight by 29%. It also raised leaf catalase activity and fruit lycopene content by 12%, and increased pulp pH and fruit firmness.	[67]
Composite of chitosan (ZnOHap@Cs) + Zinc oxide nanoparticles (ZnONPs)	Tomato (*Solanum lycopersicum* var. ‘Campbell 33’)	Auxiliary antimicrobial treatment	Increased root biomass, photosynthetic pigment content (14%), and net photosynthesis, and proved to be an effective fungicide against *Fusarium oxysporum* and *Alternaria solani*, reducing radial growth by more than 75% and 67%, respectively.	[68]
Starch-based nanocomposite natural char nanoparticles (NCNPs) hydrogel-coated NPK ^4^ fertilizer with carnauba wax/starch-latex shell.	Tomato (*Lycopersicon esculentum* Mill.)	Developing a slow-release NPK fertilizer	The slower release of NPK increased root dry weight by 93.22%, fruit dry mass by 9.52%, and total yield. It also enhanced fruit nutrient uptake (Ca 38.18%, Fe 57.46%, Zn 39.75%, K 25.32%, and P 12.69%) and raised vitamin C (63.63%), lycopene (20.52%), and gallic acid (20.4%).	[59]
Chitosan-Loaded Copper Oxide Nanoparticles Nanocomposite (CH@CuO-NPs)	Tomato (*Lycopersicon esculentum*)	Antifungal Fusarium wilt diseases (*F. oxysporum* f. sp. lycopersici (FOL))	The nanocomposite showed strong antifungal activity, reaching 96.48 ± 0.32% growth inhibition with higher concentrations of CH@CuO-NPs and reducing disease severity by up to 91.5%. It improved flowering, plant height, dry weight, defense enzyme activity, and photosynthetic pigments, leading to higher tomato production. Treated tissues showed no phytotoxicity.	[69]
biobased nanocomposite of lignin and bentonite clay mineral	Tomato (*Solanum lycopersicum*)	Sustained-release nanofertilizer for urea to increase nitrogen use efficiency.	Plant height, leaf number, and wet and dry weight increased compared to the control, along with total yield and fruit traits (weight, length, diameter), firmness, and acidity at 25% and 50% treatments. Nitrogen uptake efficiency rose to 47–88% with CRU ^5^, versus 33% in the control.	[70]
Nano DAP fertilizer + hydrogels	Tomato (*Solanum lycopersicum*)	Soil treatment: Enhancing drought resilience and sustainability by improving soil-related parameters and productivity	Nano DAP with hydrogel improved water-holding capacity (78–79%) and reduced bulk density (1.18–1.15 g/cc). It also increased soil nitrogen (199–220 kg/ha) and organic carbon (0.25–0.26%), while enhancing microbial activity and improving micronutrient availability.	[71]
Graphene + Copper nanoparticles (CuNPs) nano composite (Graphene–Cu)	Tomato (*Lycopersicon esculentum*)	Foliar inoculation/plant treatment against the pathogen *Fusarium oxysporum*	The Graphene–Cu nanocomposite delayed “vascular wilt” and reduced its severity by 29%, while increasing photosynthetic pigments and fruit production. It improved the antioxidant activity by elevating glutathione, flavonoids, anthocyanins, and GPX ^6^, PAL, and CAT ^7^ activity. It also reduced water potential by 31.7% and Fv/Fm by 32%.	[72]
Hydrogel + Rosemary extract	Tomato (*Solanum lycopersicum*)	Pesticide for the control of root-knot nematodes and the activation of defense mechanisms in tomatoes.	Reduced egg masses, galls, and total nematodes in roots in a dose-dependent manner, while increasing POX ^8^ and PFO ^9^ activity, indicating induced defense responses.	[73]
Sodium alginate-based hydrogel + CuO-NPs and ZnONPs	Tomato (*Solanum lycopersicum*)	Hydrogel as a nanofertilizer: controlled release of micronutrient s (Zn, Cu)	Gradual ion release in water and soil, with a progressive increase in leaf Cu, undetectable Zn, and improved macro- and micronutrient availability without added non-biodegradable polymers.	[74]
**Corn**				
**Material**	**Species**	**Application**	**Findings**	**Ref.**
Copper nanoparticles grafted with essential oil	Corn (*Zea mays*)	Fungicide for disease management	Reduced corn leaf blight incidence by 25–27%, increased antioxidant enzymes (β-1,3-glucanase, PAL, POX, PPO ^10^) and total phenols; Improved biomass, photosynthesis, and root development, with toxicity observed only at 1000 mg L^−1^.	[75]
Gelatin hydrogel embedded with carbon dots derived from tannic acid	Corn (*Zea mays*)	Mitigation of water stress and promotion of germination Seed coating to impart positive eustress	Increased germination by 19%, shoot length by 139%, fresh biomass by 1.24×, and dry weight by 1.06–1.82×. Photosynthesis, stomatal conductance, and transpiration rose by 1.3–1.4×. Rhizosphere TC ^11^, TN ^12^, TIC ^13^, TOC ^14^, and beneficial microbial abundance also increased.	[76]
Lignin-based hydrogel	Corn (*Zea mays*)	Mitigation of water stress under conditions of drought	Maize plants were taller, accumulated more phosphorus and sodium, and produced more biomass, with an 86% reduction in proline and 10% less electrolyte leakage under severe drought, indicating reduced water stress.	[77]
Carboxymethyl Cellulose/Nano-CaCO_3_ Hydrogel (CMC/NCC)	Corn (*Zea mays*)	Soil amendment (loamy sand) to improve water retention and total organic carbon	Hydrogel use in sandy soil improved water retention capacity, reduced root biomass, and promoted root growth, without affecting total yield.	[78]
**Chili (*Capsicum*)**				
**Material**	**Species**	**Application**	**Findings**	**Ref.**
Sodium alginate-carboxymethylcellulose (SA-CMC) hydrogel + Zinc oxide nanoparticles (ZnO-NPs), KCl, and NAA hormone	Chilli (*Capsicum annuum*)	Improved flowering and crop yield	High NAA ^15^ encapsulation efficiency (~92.53%); sustained release of NAA, Zn, and K for up to 21 days. Synergistic effect that improved yield, plant height, number of branches, fruits, and flowers.	[27]
Green-synthesized Zinc oxide nanoparticles (ZnONPs)	Chilli (*Capsicum annuum* L.)	Improved yield, nutraceutical quality, and capsaicin concentration	Foliar application of 40–50 ppm improved yield, size, and number of fruits. 30–40 ppm increased vitamin C, bioactive compounds, and antioxidant capacity, indicating improved nutraceutical quality of the fruit.	[79]
Superabsorbent hydrogel based on polysaccharides from watermelon rind waste (WPW) + zinc oxide nanoparticles ZnO-NPs	Chili pepper (*Capsicum annuum*)	Fungicide for disease management (*Fusarium wilt* and *Fusarium oxysporum*)	Up to 81.81% reduction in the incidence of wilt disease; increased chlorophyll, carotenoid, osmolyte, and phenolic levels in treated plants (indicating enhanced defense and nutritional quality	[80]
Chitosan-PVA hydrogel + Copper nanoparticles (CuNPs)	Jalapeño pepper (*Capsicum annuum*)	Eustress treatment, fruit improvement	Increase in the number and average weight of fruits, capsaicin content up to 51%, ABTS ^16^ antioxidant content 4% and DPPH ^17^ 6.6%; total phenols 5.9% and flavonoids 12.7%.	[28]
**Other Cases**				
**Material**	**Species**	**Application**	**Findings**	**Ref.**
Chitosan hydrogel beads + silica nanoparticles (MSNs) loaded with urea	General agriculture–unspecified	Prolonged release and water retention of urea	Sustained release of urea for over a month, greater than 90%, and improvement in soil water retention of 75% after 30 days.	[81]
Commercial potassium-based hydrogel + ZnO-NPs + Nitrogen fertilizer	Quinoa (*Chenopodium quinoa*)	Improve germination and plant establishment	Increased germination to 50.99% (vs. 6.49% control) and plant production to 90.33 per linear meter (vs. 14.66), along with improved plant growth.	[20]
Chitosan-Coated Alginate Matrices with Protein-Based Biostimulants	Cucumber (*Cucumis sativus*)	biodegradable slow-release fertilizer	Lower initial nutrient release with controlled leaching, improved structural stability due to the coating, no phytotoxicity, and higher germination growth with lightly coated encapsulated matrices.	[1]
Chitosan-PVA hydrogel (CS-PVA) + SeNPs ^18^	Cucumber (*Cucumis sativus*)	Improve productivity and production	Increased leaf area by 10.5–29.6%, fresh root weight by 22.4%, fruit number by 26%, and yield per plant by 34.9%. Fruit size traits were highest at 1 mg Selenium nanoparticles (SeNPs), though not significantly different.	[18]
Polyacrylamide/methylcellulose hydrogel + Calcium montmorillonite clay (MMt)	None	Controlled release of macronutrients and micronutrients, mitigation of water stress	Water absorption > 5000 times its weight. MMt increased the carrying capacity of urea and boron and slowed their release.	[81]

^1^ Calcium montmorillonite, ^2^ Phenylalanine ammonia lyase enzyme, ^3^ Pathogenesis-Related 1, ^4^ Nitrogen phosphorus potassium, ^5^ Controlled-release urea, ^6^ Glutathione peroxidase enzyme, ^7^ Catalase enzyme, ^8^ Peroxidase enzyme, ^9^ Polyphenoloxidase enzyme, ^10^ Polyphenol oxidase, ^11^ total carbon content, ^12^ total nitrogen content, ^13^ total inorganic carbon, ^14^ total organic carbon content, ^15^ Naphthalene acetic acid, ^16^ 2,2′-azino-di-(3-ethylbenzthiazoline sulfonic acid), ^17^ 2,2-Diphenyl-1-picrylhydrazyl, selenium nanoparticles, ^18^ Selenium nanoparticles.

## 5. Environmental and Agronomic Benefits

### 5.1. Water Conservation

Polymeric hydrogels and their nanocomposite variants are functional materials that combine water retention with the ability to increase nutrient availability. Their controlled water retention and release reduce losses due to percolation and evapotranspiration, maintain soil moisture in the root zone, and consequently promote more stable plant development and better, higher-quality yields [15,20].

From a structural perspective, the parameters that control water adsorption and storage in a hydrogel are crosslink density and mesh size. Higher crosslink density reduces mesh size and restricts the volume of adsorbed water, while lower density allows for greater swelling and water retention capacity [1,19,20]. However, higher crosslink density improves the mechanical integrity of the material, a critical factor for withstanding soil stresses (compaction, agricultural traffic) and maintaining functionality throughout the crop cycle [1,19]. Therefore, there is a technical compromise between maximizing water retention capacity and ensuring mechanical strength; the optimization of this balance must be tailored to the specific agronomic application.

The chemical composition of polymer chains defines their affinity for water and their exchange kinetics (adsorption/desorption). Hydrophilic functional groups—hydroxyls, carboxyls, amides, amines, and sulfonates—promote interaction with water molecules through hydrogen bonding and van der Waals forces, increasing swelling capacity and the response speed to hydrometric changes [18].

The inclusion of nanoparticles in the polymer network modifies both the microstructure and the diffusion pathways, affecting the swelling capacity depending on the nature of the polymer–NP interaction. For example, in sodium alginate–carboxymethylcellulose formulations crosslinked with CaCl_2_ and added with ZnO-NPs, KCl and naphthaleneacetic acid, different swelling behavior was observed between the formulations: one of the samples reached 31.25% swelling on the seventh day and stabilized afterwards, while another formulation showed higher swelling (202.04%) on day 21 of the experiment; the authors attribute these differences to the generation of nanopores, increased surface roughness and ionic competition (K^+^ vs. Ca^2+^) that modifies the effective crosslinking density, increasing the flexibility of the chains and water diffusion pathways [27].

In contrast, the incorporation of CeO_2_-NPs into a chitosan-benzaldehyde xerogel reduced relative swelling by increasing cross-linking density but prolonged the material’s functional capacity, observing retention for more than 18 days compared to 9 days in the xerogel without nanoparticles, demonstrating that the presence of nanoparticles can enhance mechanical stability and the duration of water function even when the magnitude of swelling decreases [36].

Likewise, studies with acrylamide hydrogels incorporating watermelon peel and ZnO-NPs have reported increases in cross-linking density and a reduction in swelling, reaffirming the dependence of the response on the type and interaction of the nanoparticles with the polymer matrix [80].

### 5.2. Reduction in Fertilizer Leaching

Fertilizer leaching in agricultural soils is a central problem associated with the intensive use of agrochemicals: the dissolution and transport of soluble nutrients by irrigation or rainwater reduces fertilizer efficiency, increases costs for producers, and causes severe environmental impacts, including eutrophication of water bodies and alterations in soil chemistry [36,82,83]. This scenario has prompted the search for management strategies that minimize leaching losses and maximize nutrient use efficiency.

The application of hydrogels is positioned as an alternative to the use of conventional fertilizers, allowing for the slow or controlled release of these nutrients. Recent studies have shown that hydrogels can reduce nitrate and phosphate leaching depending on the type of hydrogel and soil texture, significantly contributing to more efficient and sustainable agriculture [36,84].

The incorporation of nanoparticles into the polymer matrix introduces additional mechanisms that increase retention and modulate release kinetics. For example, interactions between the surface of the nanoparticles and fertilizer molecules (or functional groups of the polymer) can slow diffusion, modify polymer network relaxations, and consequently prolong nutrient release [82,85].

In a study using a benzaldehyde-modified chitosan xerogel incorporating CeO_2_-NPs, the nanocomposite formulation was found to retain more than 50% of the urea content at 15 days, compared to less than 10% for the xerogel without nanoparticles at 10 days; furthermore, sustained release extended to 30 days, suggesting a molecular interaction between CeO_2_-NPs and the matrix that slows urea efflux [36].

In parallel, formulations based on sodium alginate and carboxymethylcellulose composed of ZnO-NPs, KCl and naphthaleneacetic acid showed a slow and sustained release pattern of Zn^2+^ and K^+^ ions for at least 21 days, illustrating how the combination of polymeric matrix, nanoparticles and salts/hormones allows us to design temporal supply profiles adjusted to the demands of the crop [27].

### 5.3. Contribution to Sustainable and Resilient Agriculture

Hydrogel nanocomposites offer the potential to contribute to increased sustainability and resilience in agricultural systems by combining properties such as water retention, controlled nutrient release, and the ability to generate plant physiological responses to abiotic and biotic stress. These functions allow for reduced irrigation frequency and agrochemical dosages and mitigation of losses due to diseases and pests, thus contributing to the development of agricultural practices with a smaller environmental footprint and greater resilience to climate variability.

#### 5.3.1. Resistance to Water Stress and Drought

One of the most common problems in agriculture is facing conditions of low water availability or drought, which cause a reduction in crop growth and quality [19]. Taking advantage of water retention properties as an alternative to keeping crop soil moist, studies have been conducted to extend this retention capacity for a longer period [1].

For instance, in tomato (*Solanum lycopersicum*), the combination of Nano-DAP and hydrogel increased soil water retention from 55 ± 0.37% to 78.0 ± 0.85% in the first growing season (2022–2023), and from 56 ± 0.78% to 79.0 ± 1.22% in the second (2023–2024), representing a improvement in soil water retention compared with the untreated control [71]. In eggplant (*Solanum melongena*), a magnetic nanoparticle-grafted hydrogel (HG–MNP) showed a swelling ratio of 65.43 g g^−1^, outperforming the control hydrogel (45.54 g g^−1^) [86]. For wheat (*Triticum aestivum*), the maximum swelling capacity of Raw-ZnO-HGs reached 453.9%, nearly double that of unmodified hydrogels (230.2%) [87]. In mung bean (*Vigna radiata*), the application of NP-loaded hydrogels resulted in a 15% reduction in irrigation requirements, evidencing more efficient water use under drought stress [88].

Beyond water retention, the application of hydrogel–NPs systems enhance multiple morphological, physiological, and biochemical attributes that support plant survival and yield during water deficit. In tomato, nanocomposite hydrogels enriched with carbon nanoparticles enhanced growth indices by 22–45% and nutrient contents (P, Fe, Zn) by 16–29%, while Nano-DAP hydrogels improved soil nitrogen levels up to 220 ± 29.1 kg ha^−1^ [9]. Hydrogels containing Cu-NPs also increased stem and leaf dry biomass by 20% and root biomass by 30% [89].

In rice (*Oryza sativa*), the combined application of iron oxide nanoparticles (FeO-NPs) with hydrogels increased plant height by 34.8%, fresh weight by 40.16%, and dry weight by 31.05% under drought stress, relative to controls without nanoparticles. These effects were linked to enhanced antioxidant activity, photosynthetic efficiency, and nutrient uptake [90].

For quinoa (*Chenopodium quinoa*), the co-application of hydrogel and ZnO-NPs (7 g L^−1^) significantly improved seed germination from 6.49% to 50.99% under moisture scarcity and raised the average number of plants per meter from 14.66 (control) to 82–90 plants, indicating improved establishment and vigor [91].

Finally, in soybean (*Glycine max*), the integration of Fe-NPs within supramolecular hydrogels enhanced droplet deposition on leaves by 168.9% and NP uptake by 154.8% compared with direct NP suspensions, allowing an 80% reduction in NP dosage while still achieving a 28% increase in fresh biomass [92].

#### 5.3.2. Efficient Nutrient Delivery and Biostimulation

Sustained release and, when appropriate, stimulus-sensitive release (pH, moisture, enzymatic activity) enable the plant to receive micronutrients and phytohormones at critical times of demand, optimizing processes such as elongation, flowering, and the accumulation of compounds of interest [27]. Likewise, increases in quality parameters (e.g., sugars and secondary metabolites) reported in other studies demonstrate positive effects on the organoleptic and nutritional quality of the final product [36].

For example, CuO NP-embedded chitosan/acrylic acid hydrogels exhibited 34.2–94.8% slower Cu dissolution over seven days compared with unencapsulated CuO-NPs [84]. This delayed release improves assimilation efficiency and reduces ionic toxicity risks. In *Lactuca sativa* infected with *Fusarium oxysporum* f. sp. lactucae, exposure to CuO NP-embedded hydrogels significantly increased the content of organic acids such as malic acid, butenedioic acid, gluconic acid, and galactaric acid by 33.2–682% relative to diseased controls [84]. These organic acids play crucial roles in redox balance and nutrient mobilization—particularly malate, which acts as a key redox shuttle between chloroplasts and the cytosol through the malate valve, while gluconic acid promotes phosphorus uptake in P-deficient soils, indirectly supporting stress resistance [93].

Tests with hydrogels co-loaded with ZnO nanoparticles and naphthaleneacetic acid (NAA) showed significant improvements in chili (*Capsicum annuum*) growth compared with unloaded hydrogels, evidencing their capacity to combine nutrient delivery and hormonal regulation in a single platform [19]. The potassium release profile exhibited fluctuations over 21 days—9.79% in 24 h, decreasing to 5.48% in 7 days, rising to 16.19% in 14 days, and dropping to 2.81% in 21 days—typical of matrix-type hydrogels. Zinc release was slower and more stable, reaching only 2.73% at 24 h and 1.81% at 21 days, confirming strong retention and controlled diffusion [19]. NAA exhibited an initial burst release of 58.12% within 450 min, followed by a sustained phase up to day 14 due to diffusion–reabsorption equilibrium [19]. This dual-phase release provides an early hormonal stimulus and prolonged nutrient availability, enhancing plant growth and assimilation efficiency through a balanced and controlled delivery mechanism.

#### 5.3.3. Pest and Disease Resistance; Defense Induction

The incorporation of nanoparticles with antimicrobial or insecticidal properties into hydrogel matrices enables the localized and sustained release of protective agents, minimizing the frequency of foliar applications and reducing the environmental footprint of conventional pesticides. ZnO-NP-based hydrogel formulations impart notable antifungal activity, significantly lowering the incidence of *Fusarium oxysporum* wilt by providing between 36.35% and 81.81% protection [80]. Similarly, hydrogels embedded with CuO nanoparticles effectively suppressed *Fusarium oxysporum* f. sp. lactucae infection in lettuce [84].

In parallel, solid lipid nanoparticles (SLNs) loaded with essential oils of *Origanum vulgare* and *Thymus vulgaris*, incorporated into chitosan/PVA hydrogels, exhibited potent antifungal activity against major postharvest pathogens. The *O. vulgare* essential oil-loaded SLNs, at 18.7% *v*/*v*, reduced *Botrytis cinerea* mycelial growth by 89.5%, while *T. vulgaris* SLNs at 15% *v*/*v* achieved a 57% inhibition rate. Likewise, exposure of *Penicillium expansum* to essential oil release from SLNs resulted in 93% suppression of mycelial growth. Furthermore, spore germination was also significantly affected, with inhibition exceeding 60% for B. cinerea and ranging from 25.8% to 33.8% for *P. expansum* [94].

Beyond antifungal action, hydrogels also function as nanocarriers for insect-repellent agents. Zein-based nanoparticles encapsulating botanical repellents such as glycerol compounds demonstrated notable deterrent efficacy, with *Bemisia tabaci* repellency indices of approximately 1.1% and 1.5% for encapsulated and emulsified formulations, respectively. Moreover, these nanostructured hydrogels exhibited strong activity against *Tetranychus urticae*, achieving repellency values of 82.3 ± 6.7% for cinnamaldehyde, 86.7 ± 5.1% for eugenol, and 80.2 ± 4.1% for geraniol. The polymeric matrix provided additional stabilization and protection of the volatile compounds under environmental exposure, extending their efficacy over time and highlighting their potential for sustainable pest management strategies [95]

On a physiological level, metallic nanoparticles can function as abiotic elicitors that activate the plant’s intrinsic defense machinery. Application of Cu-NPs at low concentrations has been shown to induce mild oxidative stress, triggering antioxidant systems such as catalase (CAT) to mitigate reactive oxygen species (ROS) damage [67,96]. In Fusarium-infected lettuce, CuO-NP-loaded hydrogels improved the uptake of essential nutrients including P (21.3%), Mn (105%), Zn (57.6%), and Mg (19.7%), reinforcing both nutritional and immune status [84]. Moreover, hormonal regulation was observed through increased salicylic acid (SA) and decreased jasmonic acid (JA) and abscisic acid (ABA) levels—changes associated with enhanced systemic resistance and mitigation of xylem-related water stress.

Finally, exposure to metallic nanopartciles often enhances the biosynthesis of bioactive compounds with defensive roles. Treatments with Cu-NPs embedded in chitosan–PVA hydrogels increased the total phenolic and flavonoid contents as well as lycopene accumulation in tomato fruits, correlating with stronger antioxidant and antimicrobial defense.

### 5.4. Challenges and Future Perspectives

#### 5.4.1. Fate of Materials After Biodegradation

The degradation of hydrogels in agricultural soils is a key aspect to ensuring their sustainable use, as it prevents the accumulation of waste and determines the final fate of fertilizers, nanoparticles, and potential byproducts [21]. This process depends primarily on the chemical composition of the hydrogel, the nature and stability of the encapsulated nanoparticles, and the soil and climatic conditions that regulate microbial activity and abiotic degradation pathways.

**Figure 2:**
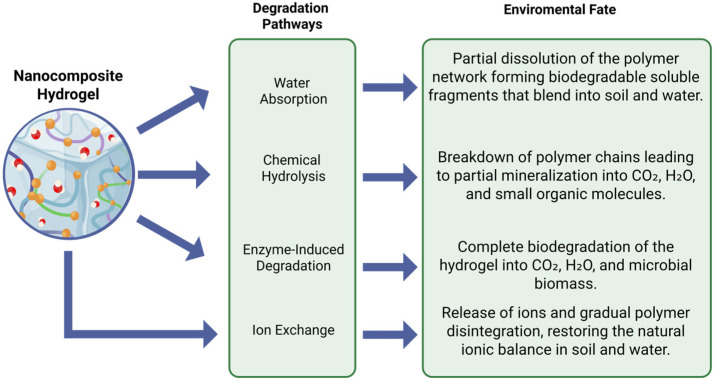
Schematic representation of degradation pathways and environmental fate of nanocomposite hydrogels in agricultural soils. A nanocomposite hydrogel with nanoparticles, water, nutrients, and diverse molecules (**left**) undergoes several concurrent degradation pathways in the soil environment (**center**), including water absorption and swelling, chemical hydrolysis of polymer chains, enzyme-induced degradation mediated by soil microbiota, and ion-exchange processes. These pathways lead to different environmental outcomes (**right**): partial dissolution of the polymer network into biodegradable soluble fragments, breakdown and partial mineralization into CO_2_, H_2_O and small organic molecules, complete biodegradation into CO_2_, H_2_O and microbial biomass, and the gradual release of ions coupled to polymer disintegration, which helps restore the natural ionic balance in soil and water.

To provide an integrated view of how these materials evolve in soil, Figure 2 summarizes the main degradation pathways of nanocomposite hydrogels and their associated environmental fate. The scheme links water absorption, chemical and enzymatic breakdown, and ion-exchange processes with the formation of soluble fragments, partial or complete mineralization, and ion release, offering a conceptual framework to interpret the experimental observations discussed in this section.

In natural hydrogels, degradation is typically mediated by the action of microorganisms and soil enzymes, which hydrolyze the glycosidic bonds and promote fragmentation of the polymer network. The porous structure of the hydrogel facilitates the infiltration of water and microorganisms, accelerating biological degradation and mineralization [24]. Therefore, porosity and mesh size are key parameters that determine both the degradation kinetics and the release of active ingredients: a more open network facilitates enzymatic access and solute diffusion, while a denser network limits both processes.

Degradation rate is a critical attribute from an agronomic perspective because it must be compatible with the crop cycle and the temporal profile of nutrient release. Reference studies show high variability: a chitosan-benzaldehyde xerogel with CeO_2_-NPs showed a weight loss of more than 90% in 60 days [36]. An alginate-CMC hydrogel composed of ZnO-NPs, KCl and naphthaleneacetic acid exhibited almost complete degradation (99.98% weight loss) at day 20, compatible with short-cycle crops such as chili [27].

Efforts at understanding the degradation of these materials could benefit from the incorporation of metabolomic approaches involving crops. This type of study would allow us to understand how the release of nutrients and nanoparticles, as well as the degradation of the polymer into microplastics, modifies the metabolic profile of crops, identify potential alterations in biochemical pathways associated with growth, plant defense, or the accumulation of compounds of agricultural interest [82].

#### 5.4.2. Potential Ecotoxicity of Released Nanoparticles

Despite the agronomic benefits of nanocomposite hydrogels, the release of nanoparticles into the environment raises significant concerns about their ecotoxicity. Metallic or semimetallic nanoparticles used to improve hydrogel functionality can interact with soil organisms, water, and non-target biota, affecting soil microbiota, nutrient uptake, and the health of aquatic ecosystems [97].

Recent studies have shown that the accumulation of silver, zinc oxide, or copper oxide nanoparticles can cause oxidative stress in microorganisms and plants, altering nutrient dynamics and soil biodiversity [57]. Therefore, it is crucial to evaluate not only the agronomic efficiency of nanocomposites, but also their fate, persistence, and potential toxicity, considering the dose, size, shape, and coating of the nanoparticles to minimize environmental risks.

The design of biodegradable hydrogels with safe nanoparticles or controlled release of bioactive agents represents a strategy to reduce adverse impacts. The integration of environmental monitoring systems and ecotoxicological testing in field trials allows for the identification of safe application limits and ensures that productivity and resilience benefits do not compromise ecosystem health [17].

#### 5.4.3. Regulatory Challenges and Field Acceptance

The implementation of nanocomposite hydrogels in modern agriculture faces multiple regulatory challenges, mainly due to the novelty of these materials and the complexity of their components. These products combine hydrophilic polymers and functional nanoparticles, placing them in a gray area within existing regulations for fertilizers, biostimulants, and pesticides. In many countries, there are no clear guidelines for assessing the environmental safety, potential toxicity, and persistence of nanocomponents, making formal approval for agricultural use difficult [2]. The lack of unified global standards also limits the international export and commercialization of these materials.

Field acceptance depends on regulatory factors as well as socioeconomic and educational aspects. Farmers need tangible evidence that nanocomposite hydrogels improve yield, crop quality, and water and nutrient use efficiency without posing environmental or health risks. Uncertainty about production costs, agronomic benefits, and safe handling can lead to resistance to adoption, especially in small-scale agricultural systems [16].

To overcome these barriers, it is recommended to implement standardized application protocols, conduct long-term environmental risk and toxicity assessments, and develop agricultural training and extension programs that facilitate the understanding and safe management of these materials. The integration of nanocomposite hydrogels into sustainable agricultural systems therefore depends not only on their technical effectiveness, but also on farmer confidence and a robust regulatory framework capable of balancing innovation, safety, and sustainability.

Taken together, these agronomic, environmental and regulatory considerations indicate that the deployment of nanocomposite hydrogels is not only a scientific and ecological challenge, but also a technological and market issue. Understanding how current products, patent-protected technologies and commercial formulations are evolving is therefore essential to assess which concepts are already close to practical implementation and where critical gaps still exist.

## 6. Technological and Market Landscape

Building on the constraints and opportunities outlined above, this section examines the current technological and market landscape of nanocomposite hydrogels for agriculture, focusing on patent activity and commercially available formulations.

### 6.1. Patents Analysis

An analysis of recent patent applications reveals a technological transition from basic nutrient delivery systems to multifunctional, bioactive platforms. A central focus of innovation addresses the low efficiency of conventional fertilizers. Key patents aim to develop controlled release systems that reduce nutrient loss via leaching and volatilization, improving nutrient use efficiency. For example, Sharma Y and coworkers (assigned to SABIC Agri Nutrients Co., Riyadh, Saudi Arabia) [98], discloses a nanoparticulate fertilizer formulation based on a biopolymer carrier (chitosan) encapsulating both a macronutrient (urea) and micronutrient nanoparticles (e.g., Cu, Mn, Si). The claims also include plant growth promoters and biostimulants, highlighting a shift toward integrated, multifunctional agronomical inputs.

Alternative approaches focus on fabrication methods. The patent presented by Mukkhopadhyay and Siddhartha S. [99], filed by the Indian Council of Agricultural Research (ICAR), describes the use of natural clay minerals (e.g., kaolinite, smectite) as nanoscale receptacles for nutrient ions (e.g., Zn^2+^, PO_4_^3−^), which can be further embedded in biodegradable polymers or hydrogels for soil application.

A different strategy is introduced by Biswas [100], which moves away from the hydrogel carrier model. This invention presents a nano NPK composite where the nanoparticle itself functions as the fertilizer. Produced via aerosol synthesis, this material is designed for foliar applications and aims to mitigate ecotoxicity by minimizing runoff and metal accumulation in edible plant tissues.

An evolution in the patent landscape is the progression toward bioactive systems aligned with the concept of eustress. The work developed by the Adolphe Merkle Institute [101], claims silica nanoparticles (SiO_2_-NPs) with dual functionality: controlled release of orthosilicic acid and induction of a plant priming effect. These nanoparticles act as hormetic eustressors, triggering defense pathways that enhance resistance to biotic and abiotic stressors.

Collectively, these patents reflect a clear technological trajectory: from passive carriers such as clays and hydrogels, through integrated macro/micronutrient platforms and standalone nanoparticulate fertilizers, toward smart, bioactive materials that modulate plant physiology via eustress-based mechanisms.

### 6.2. Market Analysis of Nanocomposite Hydrogel for Agriculture

The use of nanocomposite hydrogels used for agriculture is driven by the urgent global need for improved water and nutrient management in the face of climate change, drought, and soil degradation. The use of hydrogel became more popular in both academic and industrial domains worldwide over the past 50 years [102]. Current agricultural strategies increasingly prioritize the adoption of advanced soil amendments, notably hydrogels, which have emerged as a significant focal point. The widespread commercial accessibility of these materials offers substantial contributions to improved water conservation, soil quality enhancement, and augmented crop output.

Several commercial hydrogel formulations are currently available for agricultural applications. AQUASORB (Aquasorb 3005 K, Andrézieux, France), a cross-linked copolymer of acrylamide and potassium acrylate, is widely used due to its high water-absorption capacity and effectiveness as a soil moisture regulator across diverse cropping systems [20].

AgroNanoGel (Artagro Polska, Miechow, Poland) represents a differentiated, certified product line with strong positioning in the European market. While it offers enhanced performance, its relatively high cost and the need for field validation across soil types and climatic conditions remain key considerations. Reported lifetimes of up to five years may also vary under practical conditions [103].

AgraGel (T-400) (Terawet Green Technologies, Inc., Santee, CA, USA) consists of water-absorbing crystals capable of retaining several hundred times their weight in water. The product is pH-neutral, stable, and easy to incorporate into soil, making it a commonly used option for moisture-deficit environments [104].

Alsta Hydrogel (Chemtex Speciality Limited, Kolkata, West Bengal, India) is marketed as an eco-friendly polymer intended to reduce irrigation demand and support plant performance in arid regions. Its adaptability makes it suitable for a broad range of farming systems seeking water-use efficiency [105]. 

BountiGel^®^ (Carbon Neutral AG Sciences, Sunnyvale, CA, USA) is positioned as a high-performance amendment, with polymers capable of absorbing approximately 250 times their weight in water. It targets water-limited production systems where improved retention in the root zone can stabilize yields [106].

Currently, the market is an equation of trade-offs, where adoption is influenced by crop value, soil profile, and regional water stress. However, the overarching potential of hydrogels is severely limited by critical disadvantages: high production costs, poor field reliability due to low mechanical strength, and environmental concerns surrounding synthetic formulations.

## 7. Conclusions

Nanocomposite hydrogels represent a promising platform to advance toward more sustainable, efficient, and resilient agricultural systems. According to the studies reviewed, these materials consistently improved water management and plant performance under stress. For example, Nano-DAP hydrogels increased soil water retention in tomato from ~55–56% to ~78–79% over two seasons, Raw-ZnO hydrogels in wheat nearly doubled swelling capacity (230.2% to 453.9%), and NP-loaded hydrogels in mung bean enabled a 15% reduction in irrigation. In rice, FeO-NP hydrogels increased plant height, fresh weight, and dry weight by 34.8%, 40.16%, and 31.05%, respectively, while hydrogel–ZnO-NP systems in quinoa raised germination from 6.49% to 50.99% and plant density from 14.66 to 82–90 plants m^−1^, confirming that physicochemical improvements translate into tangible agronomic gains.

Beyond water management, nanocomposite hydrogels act as multifunctional carriers that enhance nutrient-use efficiency and biotic stress control. CuO-NP hydrogels slowed Cu dissolution by 34.2–94.8%, increased key organic acids by 33.2–682% in diseased lettuce, and improved nutrient uptake (e.g., Mn +105%, Zn +57.6%). Systems co-loaded with ZnO-NPs and NAA in chili showed controlled, phased release of K, Zn and phytohormone, while ZnO-NP hydrogels provided 36.35–81.81% protection against *Fusarium* wilt and essential-oil SLN hydrogels reduced *Botrytis cinerea* and *Penicillium expansum* growth by up to 89.5% and 93%. In soybean, Fe-NP supramolecular hydrogels increased NP uptake by 154.8% and fresh biomass by 28% while allowing an 80% reduction in NP dosage. Together, these results support a dual mechanism: the hydrogel matrix buffers water and nutrients and reduces leaching (up to 40–60% in some reports), while nano- or phytochemical components act as eustressors that modulate plant physiology, enhancing stress tolerance, yield, and nutraceutical quality.

At the same time, the environmental and translational dimensions of these technologies remain only partially resolved. The degradation of natural and synthetic matrices and the long-term fate of released nanoparticles may alter soil microbiota, nutrient cycling, and ecotoxicological risk, particularly when metallic NPs accumulate in soils or trophic chains. Existing evidence, although encouraging for short-term performance, is still fragmented regarding multi-season behavior, microplastic-like residues from synthetic SAPs, and interactions between degradation products, nanoparticles, and plant metabolism. More robust evaluations of biodegradation kinetics, NP transformation, and their impacts on soil–plant–microbe systems are therefore essential to ensure that agronomic benefits do not compromise environmental integrity.

Future research should therefore prioritize: (i) long-term, field-scale trials that quantify water savings, fertilizer-use efficiency, yield stability, and disease suppression across multiple seasons and soil types; (ii) integrative eco-toxicological studies (including metabolomics and microbiome analyses) to track how polymer degradation and NP release affect plant metabolism, soil biodiversity, and food safety; and (iii) the rational design of crop- and context-specific formulations. In high-value horticultural crops such as tomato, chili pepper, and leafy vegetables grown under irrigated or greenhouse conditions, nanocomposite hydrogels loaded with micronutrients and biostimulants are particularly promising for enhancing fruit quality and nutraceutical attributes. In contrast, for cereals (maize, wheat, rice) in arid, semi-arid, or degraded soils, formulations should prioritize water retention, mechanical robustness, and controlled macronutrient release to support drought resilience and yield stability. Saline or marginal soils will benefit most from biodegradable, bio-based hydrogels that minimize persistence and support soil restoration.

Finally, the successful integration of nanocomposite hydrogels into real-world agriculture will depend on more than technical performance alone. Techno-economic analyses, clear regulatory frameworks for nano-enabled inputs, and targeted extension programs for farmers are needed to align material design with affordability, safety, and user acceptance—especially in low-resource and smallholder systems. If these scientific, regulatory, and social challenges are addressed in parallel, nanocomposite hydrogels can evolve from promising prototypes into mature, smart delivery platforms that contribute meaningfully to food security, climate-change adaptation, and environmentally responsible crop production.

## Data Availability

No new data were created or analyzed in this study. All data discussed are taken from previously published sources that are properly cited in the manuscript.

## Abbreviations

The following abbreviations are used in this manuscript:

MPK	Monopotassium-phosphate
NPK	Nitrogen-phosphate-potassium
NC-MMt	Calcium montmorillonite
PR1	Pathogenesis-Related 1
CRU	Controlled-release urea
PAL	Phenylalanine ammonia lyase
DAP	Diammonium phosphate
GPX	Glutathione peroxidase
CAT	Catalase enzyme
NAA	Naphthalene acetic acid
POX	Peroxidase enzyme
PFO	Polyphenoloxidase enzyme
PPO	Polyphenol oxidase
TC	Total carbon content
TN	Total nitrogen content
TIC	Total inorganic carbon
TOC	Total organic carbon content
ABTS	2,2′-azino-di-(3-ethylbenzthiazoline sulfonic acid)
DPPH	2,2-Diphenyl-1-picrylhydrazyl
PAM	Polyacrylamide
PAA	Polyacrylic acid
PVA	Polyvinyl alcohol
CMC	Carboxymethylcellulose
PVP	Polyvinylpyrrolidone
NPs	Nanoparticles
